# Acute kidney injury in idiopathic membranous nephropathy with nephrotic syndrome

**DOI:** 10.1080/0886022X.2021.1942913

**Published:** 2021-06-22

**Authors:** Tianxin Chen, Ying Zhou, Xinxin Chen, Bo Chen, Jingye Pan

**Affiliations:** a Department of nephrology, The First Affiliated Hospital of Wenzhou Medical University, Wenzhou, China; b Department of ICU, The First Affiliated Hospital of Wenzhou Medical University, Wenzhou 325000, Zhejian province, P.R.China; Key Laboratory of Intelligent Critical Care and Life Support Research of Zhejiang Province

**Keywords:** Membranous nephropathy, acute kidney injury, nephrotic syndrome, chronic kidney disease, end-stage renal disease

## Abstract

**Background and objectives:**

The impact of acute kidney injury (AKI) on the progression of renal function in idiopathic membranous nephropathy (iMN) with nephrotic syndrome (NS) patients have not yet been reported, we sought to investigate the incidence, clinical features and prognosis of AKI in iMN with NS patients and determine clinical predictors for progression from AKI to advanced chronic kidney disease (CKD) stage.

**Methods:**

We analyzed clinical and pathological data of iMN with NS patients retrospectively collected from Jan 2012 to Dec 2018. The primary renal endpoint was defined as persistent eGFR <45ml/min per 1.73 m^2^ more than 3 months. Comparisons of survival without primary renal endpoint were performed by Kaplan-Meier curves and log-rank test. Univariate and multivariate Cox proportional hazard models were constructed to determine independent variables associated with primary renal endpoint .

**Results:**

434 iMN with NS patients were enrolled. The incidence of AKI 1 stage, AKI 2 stage and AKI 3 stage was 23.1, 4.8 and 0.7% respectively. 66 (53.2%) patients with AKI had complete renal function recovery and 42 (33.9%) patients with AKI reached primary renal endpoint. Survival without primary renal endpoint was worse in AKI patients than No AKI patients (67.1 ± 5.3 and 43.7 ± 7.3% vs 99.5 ± 0.5 and 92.5 ± 4.2% at 2 and 4 years,*p* < 0.001). AKI was independently associated with primary renal endpoint, with an adjusted hazard ratio(HR) of 25.1 (95%CI 7.7–82.1, *p* < 0.001).

**Conclusions:**

AKI was usually mild and overlooked in iMN patients with NS, but it had a strong association with poor clinical outcomes and was an independent risk factor for CKD progression.

## Introduction

Idiopathic membranous nephropathy (iMN) is one of the most common glomerular pathological types of primary nephrotic syndrome (NS) [1,2], which also was the most frequent biopsy finding and the leading cause of NS in Chinese patients aged >40 years [3]. 10–20% of iMN patients may progress to end-stage renal disease (ESRD) [4]. Patients with iMN are significantly burdened with high disease severity and adverse health outcomes, resulting in substantial health care resource utilization and costs [5]. Many reports focused on acute kidney injury (AKI) in NS patients in recent years [6–9]. Our previous study showed 95 (34%) patients of all 277 primary NS had AKI [10]. However, there are limited data on the incidence of AKI and the impact of AKI on the progression of renal function in iMN with NS patients. A better understanding of this condition will help improve patient outcomes. This study aims to investigate the incidence, clinical features and prognosis of AKI in iMN with NS patients and evaluate factors that may affect the renal outcomes.

## Materials and methods

### Patient population

Patients with primary NS and biopsy-proven MN were reviewed retrospectively in the nephrology department of the First Affiliated Hospital of Wenzhou Medical University from Jan 2012 to Dec 2018. This retrospective observational study was approved by institutional review boards (Issuing Number 2021035), with a waiver of the need for an informed consent. Data were collected anonymously. The entry criteria: patients fit the clinical diagnosis of primary NS and renal pathological diagnosis of MN, had follow-up for at least three months. The exclusion criteria: patients with chronic renal insufficiency; secondary NS (Lupus nephritis, Sjogren's syndrome, hepatitis B virus associated glomerular nephritis, Tumor); MN combined with diabetic nephropathy, IgA nephropathy, focal segmental glomerular sclerosis (FSGS), proliferative glomerular nephritis, focal necrosis nephritis, cryoglobulinemia; AKI caused by hypovolemia, renal vein thrombosis, post-renal obstruction or acute interstitial nephritis.

### Definition

Classification and diagnosis of AKI: The change in serum creatinine (Scr) level was used to diagnose and classify AKI stage according to the Kidney Disease: Improving Global Outcomes (KDIGO) AKI criteria [11]: Diagnosis of AKI was increase in Scr by X26.5 μmol/l within 48 h or Increase in Scr to X1.5 times baseline within the prior 7 days; AKI 1stage was increase in Scr to 1.5–1.9 times baseline; AKI 2 stage was increase in Scr to 2.0–2.9 times baseline; AKI 3 stage was increase in Scr to 3.0 times baseline. The mean Scr value of patients without AKI was used as the baseline value for patients without basic Scr levels. Community-acquired AKI (CA-AKI) was defined as patients whose Scr was elevated to meet KDIGO AKI criteria on the first day of hospital admission. Hospital-acquired AKI (HA-AKI) was defined as an increase in Scr that occurred twenty-four hours or longer after hospitalization. NS was defined as the presence of proteinuria in excess of 3.5 g/d and serum albumin less than 30 g/L with or without edema and hyperlipidemia. Acute tubular injury (ATI) on renal biopsy was defined by the presence of tubular simplification, loss of brush border and enlarged reparative nuclei with or without mitotic figures. Glomerular basement membrane (GBM) was classified into four stages [12].

### Data collection and follow-up

Demographic, clinical, pathological and laboratory data were retrieved from the electronic records system of our hospital. Clinical data included medical history, physical examination and diagnosis. Pathological data included light microscopic, immunofluorescent and electronic microscopic examination. Laboratory data included Scr, serum albumin, total cholesterol (TC), Triglycerides (TG), low-density lipoprotein (LDL), hemoglobin (Hb), 24-h proteinuria. Estimated glomerular filtration rate (eGFR) was calculated by the chronic kidney disease epidemiology research group (CKD-EPI) equation [13]. Scr was measured at least once weekly in hospital. After patients improved clinically and discharged from hospital, follow-up visits were carried out in outpatient service and Scr was measured monthly. Patients would have follow-up for at least three months after discharge from hospital.

### Study end point

The primary renal endpoint was defined as persistent eGFR < 45 mL/min per 1.73 m^2^ more than 3 months. Secondary end points were NS remission and renal function recovery. Complete remission: Urinary protein excretion <0.3 g/d, confirmed by two values at least one week apart, accompanied by a normal serum albumin level, and a normal Scr. Partial remission: Urinary protein excretion < 3.5 g/d and a 50% or greater reduction from peak values confirmed by two values at least one week apart, accompanied by an improvement or normalization of the serum albumin level and stable Scr [14]. Complete renal function recovery was defined as the return of decreased renal function to pre-AKI baseline levels.

### Statistical methods

Normally or near normally distributed continuous variables were expressed as means ± standard deviation (SD), and compared using Student’s t-test. Categorical data are expressed as percentages and tested using the chi-square test. Comparisons of survival without primary renal endpoint were performed by Kaplan-Meier curves and log-rank test. Univariate and multivariate Cox proportional hazard models were constructed to determine independent variables associated with primary renal endpoint. Results were expressed as a hazard ratio (HR) with 95% confidence intervals (CIs). All statistical tests were two-tailed; *P* values less than 0.05 was considered statistically significant. Data were analyzed using the SPSS version 16 (SPSS, Inc., Chicago, IL, USA).

## Results

### Incidence of AKI in iMN patients with NS

**Figure 1.**
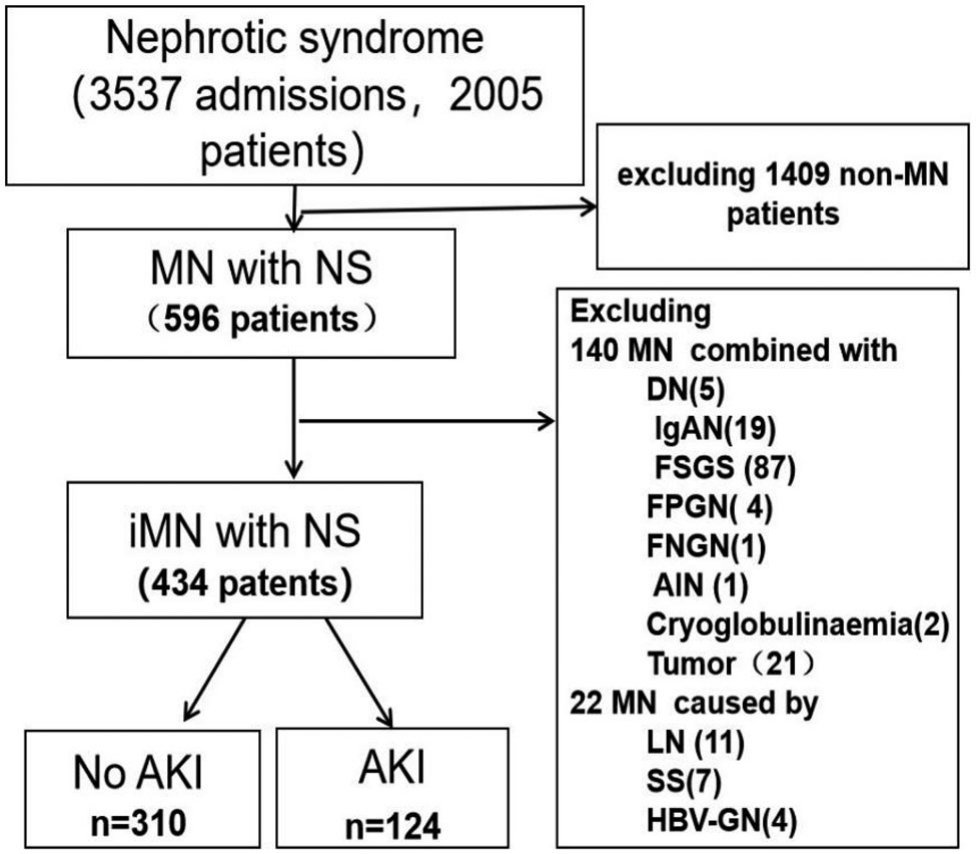
Description of patients selection. MN: membranous nephropathy; iMN: idiopathic membranous nephropathy; NS: nephrotic syndrome; DN: Diabetic nephropathy; IgAN: IgA nephropathy; FSGS: focal segmental glomerular sclerosis;FPGN: focal proliferative glomerulonephritis; FNG: Focal necrosis glomerulonephritis; AIN,acute interstitial nephritis; AKI, acute kidney injury; LN: lupus nephritis; SS: Sjogren's syndrome; HBV-GN: hepatitis B virus associated glomerular nephritis.

Over the 7-year study period, there were 3537 admissions and 2005 patients with NS. 596 NS patients were biopsy-proven MN. 162 MN patients were excluded. At last, 434 iMN with primary NS were enrolled (Figure 1), including 265 (61.1%) males and 169 (38.9%) females ranging in age from 18-84 years with a mean age of 52.3 ± 13.4 years. The mean follow-up duration after admission was 20.8 ± 15.8 months, ranging from 3 to 84 months. 310 (71.4%) patients were no AKI (No AKI group) and 124 (28.6%) patients had AKI (AKI group) . On the basis of KDIGO AKI class criteria [11], a total of 23.1% (*n* = 100) of iMN with NS patients met the stage 1 criteria, 4.8% (*n* = 21) met the stage 2 criteria, 0.7% (*n* = 3) met the stage 3 criteria, and no one required renal replacement therapy; 26 (6.0%) patients had CA-AKI and 98 (22.6%) patients had HA-AKI.

### Demographics and clinical characteristics of the study cohort

The demographics and clinical characteristics of study cohort are shown in Table 1. There were no significant differences in gender, diastolic blood pressure (DBP), TC, TG and LDL between two groups at baseline; however, age, SBP, hemoglobin (Hb), proteinuria, Scr and serum albumin at baseline were significantly different between two groups. AKI group was older and had higher SBP, lower serum albumin and more proteinuria compared with No AKI group.

**Table 1.** Baseline clinical characteristics.

Characteristic	No AKI	AKI	*p* Value
N	310	124	
male, n(%)	182 (59)	83 (67)	0.112
Age(yr)	50 ± 14	57 ± 12	<0.001
SBP(mmHg)	135 ± 20	143 ± 25	<0.001
DBP(mmHg)	81 ± 12	82 ± 13	0.394
Hb(g/L)	129 ± 18	125 ± 19	0.066
TC(mmol/L)	8.1 ± 2.5	8.3 ± 2.8	0.568
TG(mmol/L)	2.9 ± 2.1	3.2 ± 2.6	0.173
LDL(mmol/L)	4.9 ± 2.1	4.9 ± 2.3	0.998
Salb(g/L)	21.5 ± 4.3	20.3 ± 4.2	0.005
Salb ≤ 15g/l, n(%)	15 (4.8)	7 (5.6)	0.729
Scr(μmol/L)	67.2 ± 16.5	82.9 ± 32.2	<0.001
eGFR(ml/min/1.73m^2^)	109 ± 29	90 ± 30	<0.001
Upro(g/d)	6.1 ± 3.2	7.0 ± 3.6	0.014

SBP: systolic blood pressure; DBP: diastolic blood pressure; Hb: hemoglobin; TC: total cholesterol; TG: Triglycerides; LDL: low-density lipoprotein; Salb: serum albumin; eGFR: evaluated glomerular filtration rate; Upro: proteinuria.

### Renal pathology

Renal biopsies had been performed in all patients. The proportions of patients with GBM I, II and III stage were no differences between two groups (Table 2). There were more patients with ATI (pathological changes of ATI showed in Supplemental Figure 1) in AKI group than No AKI group at the time of renal biopsy (9.7% vs 2.2%,*p* < 0.001).The proportions of patients with chronic tubular lesions (CTL) were very low in two groups (Table 2). In the remainder of biopsy specimens, no specific changes for AKI other than the underlying disease were identified.

**Table 2.** Renal histological findings.

Pathology	No AKI	AKI	*p* Value
GBM stage			
I, *n*(%)	198 (63.9)	90 (72.6)	0.082
II, *n*(%)	107 (34.5)	34 (27.4)	0.153
III, *n*(%)	5 (1.6)	0 (0)	0.155
ATI, *n*(%)	7 (2.2)	12 (9.7)	<0.001
AAS, *n*(%)	81 (26.1)	25 (20.2)	0.191
CTL, *n*(%)	5 (1.6)	1 (0.8)	0.516

GBM: glomerular basement membrane; ATI: acute tubular injury; AAS: afferent ateriole sclerosis; CTL: chronic tubulointerstitial lesions.

### Treatment after admission

There were no significant differences of the proportion of patients received corticosteroids and cyclophosphamide between two groups. The proportions of patients received tacrolimus, cyclosporin A (CsA), and diuretics were significantly higher in AKI group than No AKI group (Table 3).

**Table 3.** Treatment received during follow-up.

Treatment	No AKI	AKI	*p* Value
Corticosteroids, *n*(%)	166 (53.5)	67 (54.0)	0.927
CTX, *n*(%)	55 (17.7)	15 (12.1)	0.149
FK506, *n*(%)	48 (15.5)	40 (32.2)	<0.001
CsA, *n*(%)	27 (8.7)	20 (16.1)	0.025
RSAI, *n*(%)	299 (96.5)	112 (90.3)	0.010
Diuretics, *n*(%)	56 (18.1)	40 (32.2)	0.001

CTX: cyclophosphamide; FK506: tacrolimus; CsA: cyclosporin A; RSAI: renin angiotesin system inhibitors.

### Renal outcomes

#### Renal function recovery

**Figure 2.**
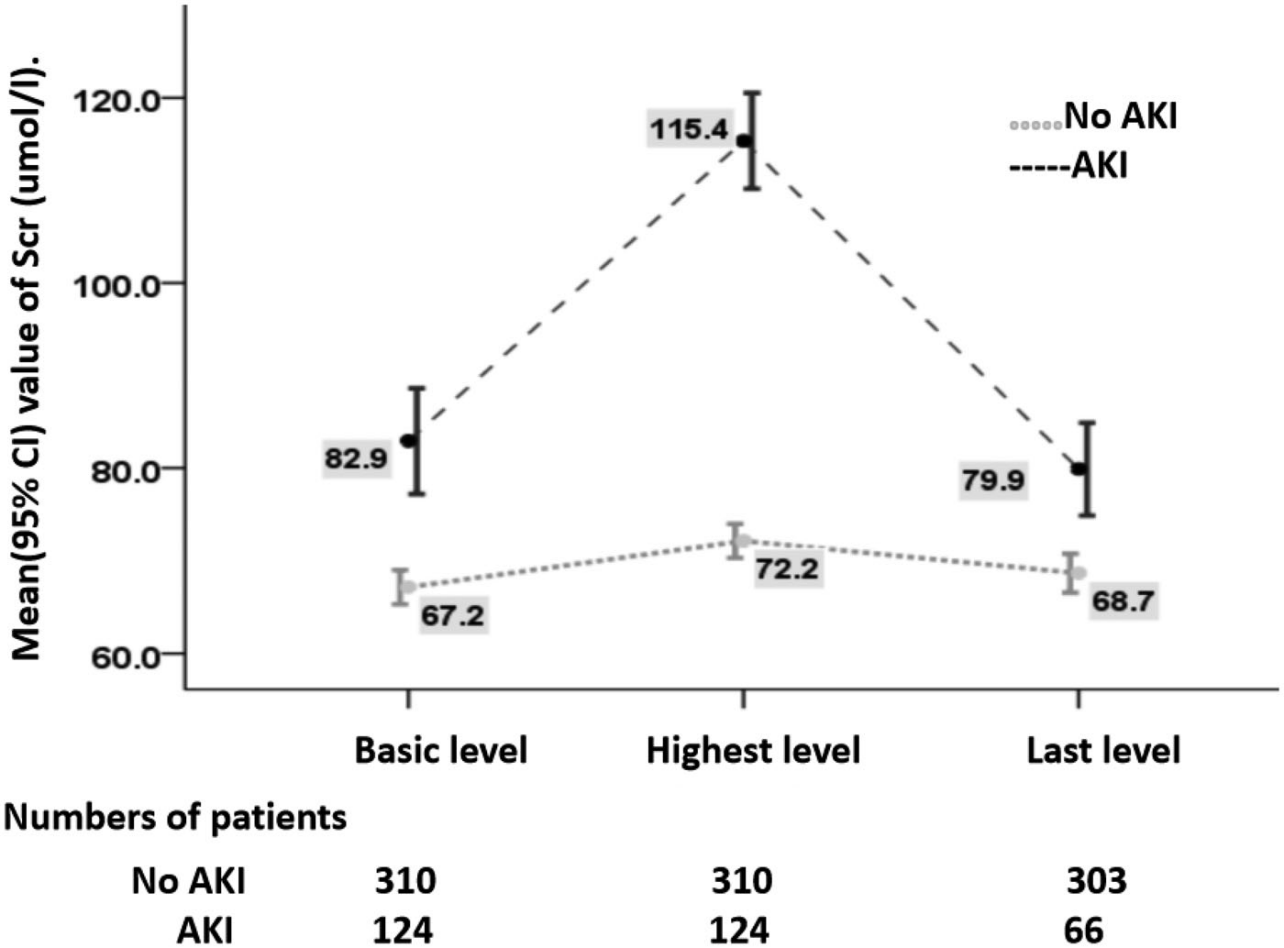
An illustration of the change of Scr in patients without renal function progression. The numbers of patients from whom readings were taken at each point are presented; variation in numbers was due to deterioration of renal function in some patients at the point.

66 (53.2%) patients in AKI group had complete renal function recovery. 16 patients without renal function recovery in AKI group progressed slowly and 42 patients in AKI group reached primary renal endpoint. 303 patients in No AKI group had normal renal function and 7 reached primary renal endpoint at the end of followup. The Scr changes of patients without chronic renal function progression in two groups are shown in Figure 2.

#### Clinical remission in two groups

On the basis of KDIGO NS remission criteria, 118 (27.2%) patients entered complete remission, 107 (24.6%) entered partial remission, and 209 (48.2%) had no remission during the course of the study. Complete remission rate was significantly lower for AKI patients (20.2%) compared with No AKI patients (30.0%, *p* = 0.037). There was no difference for partial remission between two groups.

#### Primary renal endpoint of two groups

**Figure 3.**
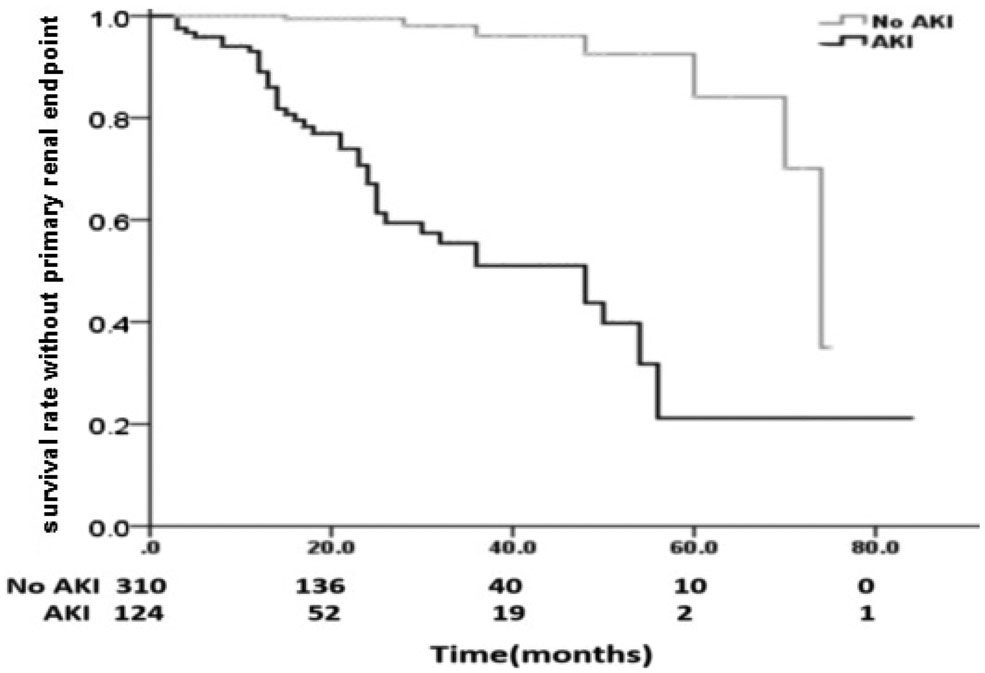
Survival rate without primary renal endpoint. Survival rate without primary renal endpoint (AKI vs No AKI) was 67.1 ± 5.3 and 43.7 ± 7.3% vs 99.5 ± 0.5 and 92.5 ± 4.2% at 2 and 4 years (*p* < 0.001); the median time to survival without primary renal endpoint was 48.0 ± 10.0 vs 74.0 ± 3.0 months.

Survival without primary renal endpoint was worse in AKI group than No AKI group (67.1 ± 5.3 and 43.7 ± 7.3% vs 99.5 ± 0.5 and 92.5 ± 4.2% at 2 and 4 years, *p* < 0.001) and the median time to survival without primary renal endpoint for group AKI and No AKI was 48.0 ± 10.0 and 74.0 ± 3.0 months respectively (*p* < 0.001) (Figure 3).

**Figure 4.**
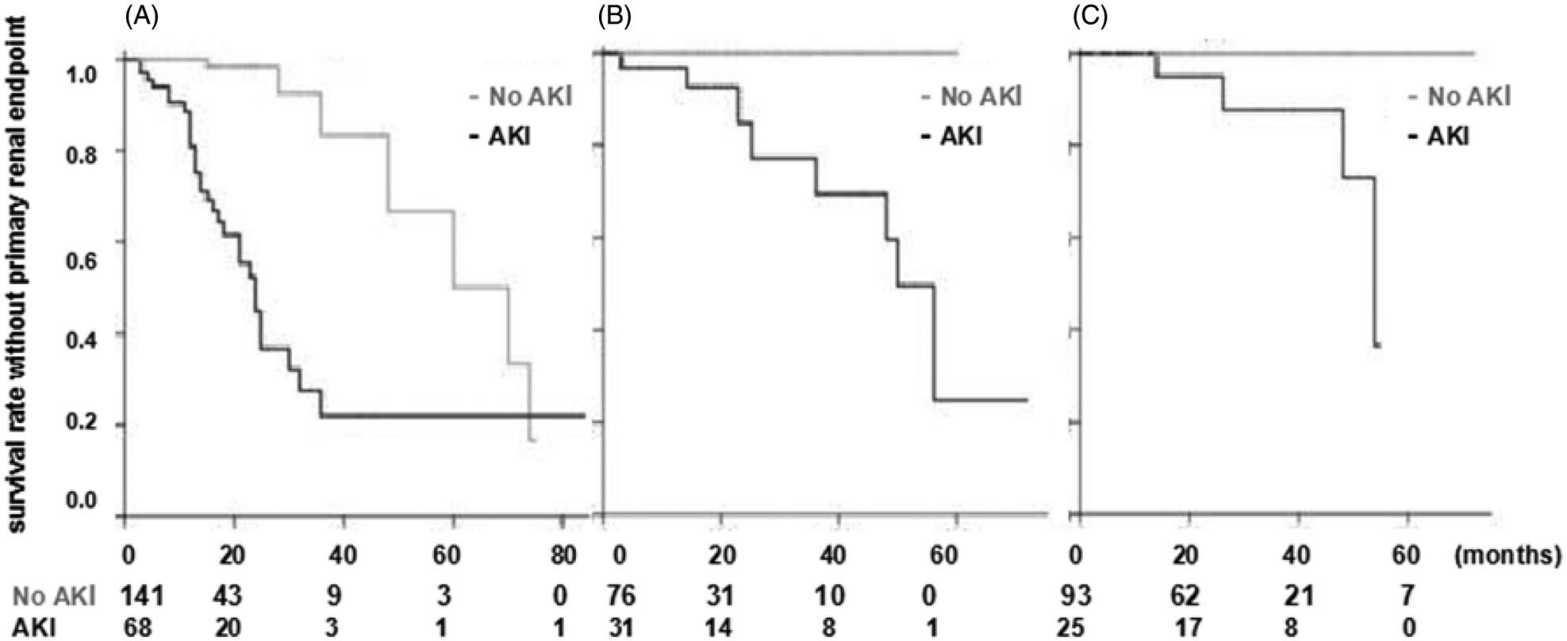
comparison of survival rate without primary renal endpoint between two groups based on remission status. (A) survival rate without primary renal endpoint (AKI vs No AKI ) was 44.9 ± 8.2% vs 98.4 ± 1.6% (*p* < 0.001) at 2 years in the cohort without remission; (B) survival rate without primary renal endpoint (AKI vs No AKI) was 84.9 ± 8.7% vs 100% (*p* = 0.001) at 2 years in the cohort with partial remission; (C) survival rate without primary renal endpoint (AKI vs No AKI) was 95.0 ± 4.9% vs 100% (*p* < 0.001) at 2 years in the cohort with complete remission.

In order to find out whether or not the renal outcome of remission patients also was affected by AKI, primary renal endpoint between two groups based on remission status was analyzed. Survival rate without primary renal endpoint at 2 years between AKI and No AKI group was 44.9 ± 8.2% vs 98.4 ± 1.6% (*p* < 0.001) in no remission patients, 84.9 ± 8.7% vs 100% (*p* = 0.001) in partial remission patients, and 95.0 ± 4.9% vs 100% (*p* < 0.001) in complete remission patients (Figure 4).

The significance of each factor affecting the primary renal endpoint is shown in Table 4. Among the clinical parameters, old age (≥55yrs), SBP ≥135mmHg, massive proteinuria (≥5.0 g/day) and AKI were significant risk factors for primary renal endpoint by univariate Cox regression analysis. In multivariate cox analysis of the clinical and pathological variables that were correlated with primary renal endpoint, AKI was independently associated with primary renal endpoint, with an adjusted hazard ratio (HR) of 25.1 (95%CI 7.7–82.1, *p* < 0.001). male (HR = 2.5, 95% CI 1.1-5.3, *p* = 0.037) and proteinuria ≥5.0 g/d (HR = 2.6, 95% CI 1.3–5.5, *p* = 0.008) were also the risk factors to predict primary renal endpoint. Patients with GBM stage II/III in renal histopathology did not have higher risk of primary renal endpoint compared with patients with GBM stage I (HR = 0.5, 95% CI 0.3–1.2, *p* = 0.130).

**Table 4.** Univariate and multivariate Cox analysis of risk factors affecting renal events.

Risk factors	Univariate Cox analysis				multivariate Cox analysis			
	HR	95%CI		*p* Value	HR	95%CI		*p* Value
Age ≥55yr	2.4	1.3	4.3	0.004	1.5	0.8	3.1	0.210
Male	1.6	0.9	2.9	0.140	2.5	1.1	5.3	0.037
AKI	14.5	6.5	32.3	<0.001	25.1	7.7	82.1	<0.001
Salb ≤15g/l	1.0	0.2	4.0	0.960	0.9	0.2	3.8	0.858
GBM stage(II or III)	1.4	0.8	2.6	0.241	0.5	0.3	1.2	0.130
SBP ≥135mmHg	2.6	1.4	5.0	0.004	2.0	1.0	3.9	0.051
Upro ≥5g/d	2.5	1.3	4.6	0.006	2.6	1.3	5.5	0.008

AKI: acute kidney injury; GBM: glomerular basement membrane; Salb: serum albumin; SBP: systolic blood pressure; Upro: proteinuria.

## Discussion

Studies in the past decade have dramatically improved understanding of the pathogenesis, clinical features and outcomes of iMN [15–20]. However, to the best of our knowledge, there were no clinical studies to investigate the epidemic and outcome of AKI in iMN with NS patients except sporadic case reports.

In our study, overall incidence of AKI was 28.6%; of these 23.1, 4.8, and 0.7% of iMN with NS patients had KDIGO AKI stages 1, 2, and 3, respectively. AKI incidence in this study was similar with the results of NS with MCD patients reported in previous studies, but the proportion of AKI-2 or AKI-3 stage in iMN was obviously lower than that in MCD [6,9,10]. 68(9.5%) of 716 iMN patients with eGFR < 60 mL/min per 1.73 m^2^ was considered as CKD ≥ 3 stage in a recent retrospective study [21], which did not differentiate AKI from CKD in patients with low baseline eGFR. In our study, AKI at initial presentation (CA-AKI) was observed only in 26 (6.0%) patients, but most patients (22.6%) developed AKI after admission and medications. Causes such as hypovolemia, renal vein thrombosis and acute interstitial nephritis were exclude in our study. Compared with No AKI patients, AKI patients were older, had higher SBP, lower serum albumin and more proteinuria. Furthermore, more AKI patients received tacrolimus, CsA, and diuretics than No AKI patients. AKI in iMN with NS may be associated with the severe clinical manifestations and high exposure to nephrotoxic medications.

Our work revealed several novel findings. Our results suggest AKI may affect remission of NS in iMN patients. Complete remission rate was obvious lower in AKI patients than No AKI patients.Some studies also showed similar results that severe AKI needed more time to remission in NS patients [6, 8–10]. Complete renal function recovery rate was only 53.2% in this study, which was obviously lower than in MCD patients in previous reports [8–10]. AKI is no longer considered to be benign, but rather an independent predictor of mortality and an important contributor to CKD [22–24]. There is however no data on the link between AKI and subsequent development or progression of CKD in iMN patients. Our study demonstrated that AKI was associated with worse chronic renal outcomes in iMN with NS patients. eGFR <45 mL/min per 1.73 m^2^ is accepted as a valid endpoint, because it is an intermediate step on the common pathway to ESRD and represents a marked loss of renal function that is highly predictive of the subsequent development of chronic renal failure. So persistent eGFR <45 mL/min per 1.73 m^2^ more than 3 months was defined as primary renal endpoint in our study. Survival rate without primary renal endpoint in AKI patients was 67.1 ± 5.3 and 43.7 ± 7.3% at 2- and 4-year, which was lower significantly than that in No AKI patients (99.5 ± 0.5 and 92.5 ± 4.2%) . About 15% of AKI patients with partial remission and 5% of AKI patients with complete remission reached primary renal endpoint at 2-year in our study. However, normal renal function was preserved in all No AKI patients with remission of NS. Renal dysfunction and heavy proteinuria have been identified as the risk factors for progression to ESRD in iMN patients in many studies [2, 19, 25–27], which did not differentiate acute from chronic renal dysfunction. Our study found AKI was a stronger predictor of primary renal endpoint by univariate and multivariate Cox regression analysis. We also found age ≥55yr, SBP ≥135mmHg and Upro ≥5g/d were univariate risk factors in iMN with NS patients.

No specific pathological changes were found to be related to AKI except for ATI. Most renal biopsies were not performed at the time of the episode of AKI, which made it difficult to identify specific pathological changes related to the development of AKI. The predictive value of FSGS for worse long-term renal outcome in IMN patients remains debated [28–30]. S Troyanov et al. showed that histological findings by light microscopy (FSGS or chonic tubulointerstitial lesions) in MN were more closely linked to preexisting and nonmodifiable factors such as age and sex rather than to the rate of renal function deterioration [26]. In order to avoid the bias in renal function decline caused by chronic histological lesions or idiopathic FSGS combined with iMN, iMN patients with FSGS were excluded in our study. The proportions of patients with chronic tubulointerstitial lesions in two groups were really low not even 2%, which was much lower than that in iMN patients in previous reports [30,31]. The low chronic pathological changes further strengthened our opinion that renal function decline was acute but not chronic injury.

Another novel finding was chronic progression of renal function in iMN patients may not actually caused by global glomerulosclerosis but by sub-acute tubulointerstitial lesions that fail to recover from AKI. Although The recent studies on the role of circulating antibodies recognizing podocyte-specific antigens have clearly identified iMN as an autoimmune disease [32–34], the pathogenesis of renal function progression in iMN remains incompletely defined. Chronic progression of renal function in IgA nephropathy usually parallels with glomerular sclerosis and progressive degrees of tubular atrophy and interstitial fibrosis. However, there was no relationship seen between severity of global glomerulosclerosis and renal function decline in iMN patients. Repeat biopsy of 52 iMN patients after 6–72 months only showed GBM changed stage from early to late in a previous report [30]. Interstitial fibrosis may be a consequence of interstitial edema which can develop during an episode of acute renal failure.

Our findings may help clinicians identify patients at high risk for rapid progression in iMN, which in turn can inform early immunosuppressive therapy (IST). For decades, clinicians have treated iMN with NS with potentially toxic IST. Most current therapeutic guidelines recommend initiating IST in iMN patients with NS resistant to supportive care after 6 months because there was no way to distinguish patients with poor outcome from those with spontaneous remission. Our study suggests iMN patients with AKI should be considered for immediate IST without waiting 6 months on supportive care alone. Many clinicians prefer to initiate therapy with CNIs to avoid the more severe adverse reaction caused by alkylating agents and high-dose steroids. CNIs may not be a good choice for iMN patients with AKI. A recent randomized controlled trial showed that CNIs were no effect in preventing renal function deterioration in iMN patients with deceased creatinine clearance (50 mL/min) [35].

This study has several limitations. First, this was a retrospective observational study from a single center. Therefore, the interpretation might be biased owing to selection error because we excluded records of iMN patients with FSGS. Future prospective studies to confirm our findings of the incidence of AKI in iMN with NS, and its immediate and long-term outcome will be required. Second, there was no standardized regimen, and the treatment decisions were dependent on the preference of individual nephrologists. Therefore, these fundamental restrictions could not be avoided in the evaluation of long-term outcomes. Third, we did not measure antibodies to the M-type phospholipase A2 receptor (PLA2R). So we could not evaluated the HR of AKI by adjusting the value of PLA2R antibody.

In conclusion, this study demonstrated the incidence clinical features and renal outcomes of AKI in iMN with NS patients. Specifically, we found AKI was an independent risk factor for CKD progression in iMN patients with NS. This finding highlights the need of distinguishing AKI from No AKI to evaluate renal function progression of iMN patient in clinical practice.

## Supplementary Material

Supplemental Material
