# Supplementary material for: Acute kidney injury in idiopathic membranous nephropathy with nephrotic syndrome
Source: Ren Fail. 2021 Jun 22;43(1):1004–11. doi: 10.1080/0886022X.2021.1942913 (PMC8231360; doi:10.1080/0886022X.2021.1942913)
Supplement: Supplemental Material [file IRNF_A_1942913_SM3161.pdf]

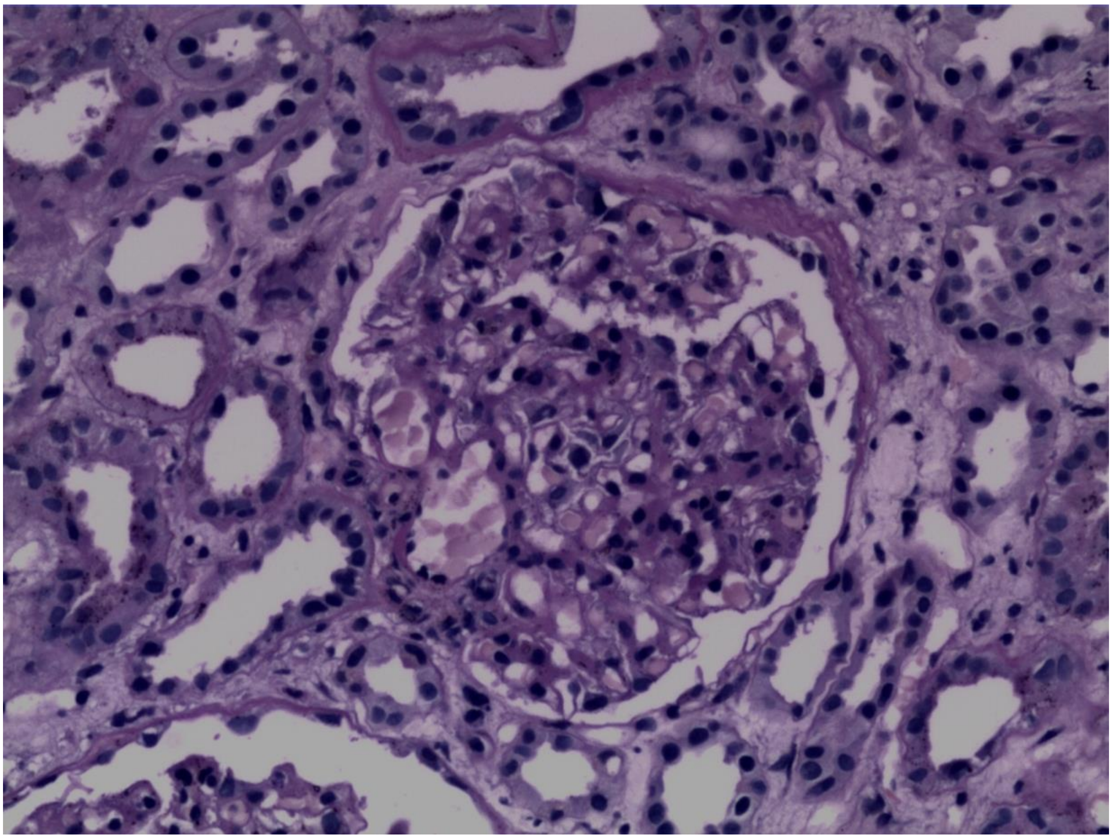

Supplemental Figure 1. Acute tubular injury (ATI) in MN with NS. ATI manifested as tubular simplification, loss of brush border, enlarged reparative nuclei and vacuolar degeneration in tubules.
